# Plasma sphingosine 1-phosphate concentrations and cardiovascular autonomic neuropathy in individuals with type 2 diabetes

**DOI:** 10.1038/s41598-020-69566-y

**Published:** 2020-07-29

**Authors:** Min Young Chung, Seon-Young Park, Jin Ook Chung, Dong Hyeok Cho, Dong Jin Chung

**Affiliations:** 1 Division of Endocrinology and Metabolism, Department of Internal Medicine, Chonnam National University Medical School, 8 Hak-Dong, Dong-Gu, Gwangju, 501-757 Republic of Korea; 2 Division of Gastroenterology and Hepatology, Department of Internal Medicine, Chonnam National University Medical School, 8 Hak-Dong, Dong-Gu, Gwangju, 501-757 Republic of Korea

**Keywords:** Diseases, Endocrinology

## Abstract

The aim of this study was to test the hypothesis that plasma sphingosine 1-phosphate (S1P) levels are associated with the risk of cardiovascular autonomic neuropathy (CAN) in type 2 diabetes patients. This cross-sectional study included 287 individuals with type 2 diabetes. CAN was evaluated using cardiovascular reflex tests. Logistic regression analyses were conducted to assess the relationship between plasma S1P levels and CAN. Plasma S1P concentrations were significantly lower in individuals with CAN than in those without CAN. There was a significant interaction between plasma S1P levels and sex with respect to CAN (*p* for interaction = 0.003). When stratified by sex, the association between plasma S1P levels and CAN exhibited a sex difference; in multivariable analysis, plasma S1P levels were significantly associated with CAN in women (odds ratio per standard deviation increase in the log-transformed value, 0.40; 95% confidence interval, 0.23–0.70, *p* = 0.001). However, there was no significant association between plasma S1P and CAN in men. Plasma S1P concentrations were inversely associated with CAN only in women with type 2 diabetes.

## Introduction

Cardiovascular autonomic neuropathy (CAN) is a common but overlooked complication of diabetes mellitus (DM)^1^. CAN involves damage to the autonomic nervous system that innervate the heart and blood vessels^2^, and it is thought to be implicated in silent myocardial ischemia, hemodynamic instability, stroke, cardiovascular mortality, and perioperative cardiovascular liability in individuals with type 2 DM^2,3^. While poor glycemic control, diabetes duration, and hypertension play important roles in the pathogenesis of CAN^4^, CAN risk cannot be entirely explained by these conventional risk factors, suggesting that other factors may also have an influence on its pathogenesis.

Sphingosine 1-phosphate (S1P), a metabolite of sphingolipids, is a bioactive lipid mediator which regulates cell differentiation, proliferation, apoptosis, and inflammation^5^. Accumulating evidence indicates that S1P plays important physiologic roles in the central and peripheral nervous systems^6,7^. Furthermore, S1P plays a crucial role in neural development^6^. In addition, several investigations have shown that decreased S1P levels may be related to neurodegenerative disease, suggesting that it also plays a neuroprotective role^8^. However, although S1P has been implicated in neurological disorders, the relationship between S1P and CAN in individuals with type 2 DM has not been clarified.

In the present study, we tested the hypothesis that plasma S1P levels are associated with CAN risk in individuals with type 2 DM.

## Methods

### Study population

This cross-sectional study included 287 individuals with type 2 DM. We consecutively enrolled individuals with type 2 DM who visited the diabetes clinic at our hospital. Type 2 DM was diagnosed based on the “Report of the Expert Committee on the Diagnosis and Classification of Diabetes Mellitus”^9^. Individuals were regarded as having hypertension if they had a blood pressure ≥ 140/90 mmHg or took antihypertensive drugs. Hyperlipidemia was considered as total cholesterol level ≥ 6.5 mmol/L and/or triglyceride level ≥ 2.3 mmol/L, or the use of lipid-lowering drugs. We gathered demographic information on smoking status, diabetes duration, and other health factors using standardized inquiries. For women, menopausal status was considered according to self-reported cessation of menses for ≥ 12 consecutive months not due to pregnancy or medical treatment. The exclusion criteria of this study included a history of glucocorticoid use, advanced renal dysfunction (serum creatinine levels ≥ 176 μmol/l), liver cirrhosis, pancreatitis, thyrotoxicosis, hypothyroidism, arrhythmia, heart failure, respiratory distress, alcoholism, infection, or malignancy. The study was approved by the ethics committee of Chonnam National University Hospital, and informed consent was obtained from all participants. The study was performed according to the Helsinki Declaration-based ethical principles for medical research involving human subjects.

### Methods

After fasting overnight, venous blood samples were collected from the participants. Glycated Hb (A1C) was analyzed by ion exchange liquid chromatography using an HLC-723-GHbV analyzer (Tosoh, Tokyo, Japan). Serum total cholesterol, triglyceride, low-density lipoprotein cholesterol (LDL-C), and high-density lipoprotein cholesterol (HDL-C) levels were determined using an AU5400 analyzer (Olympus, Tokyo, Japan). S1P levels were determined using commercially available competitive enzyme-linked immunosorbent assay (ELISA) kits (Echelon Biosciences Inc., Salt Lake City, UT) according to the manufacturer’s instructions^10^. The intra- and interassay coefficients of variations were 6.5% and 8.4%, respectively. Albuminuria was identified on the basis of the urine albumin-to-creatinine ratio (UACR) using random urine samples. Estimated glomerular filtration rate (eGFR) was calculated using the Chronic Kidney Disease Epidemiology Collaboration equation^11^. Nephropathy was defined as a UACR ≥ 300 mg/gCr or eGFR < 60 ml/min/1.73m^2^. After the patients’ pupils were dilated, fundoscopy was performed to evaluate diabetic retinopathy. Cardiovascular autonomic dysfunction was assessed by determining heart rate (HR) responses to postural change, deep breathing, and the Valsalva maneuver^1,12^. To evaluate the HR responses to standing, the ratio of the 15th R-R interval to the 30th R-R interval was calculated; a ratio below 1.00 was considered abnormal^1,12^. We assessed the beat-to-beat variation in HR during paced deep breathing by estimating the ratio of the shortest R-R interval during inspiration to the longest R-R interval during expiration, and compared this to age-related reference values^1,12^. To evaluate HR responses during the Valsalva maneuver, the subject forcefully exhaled through the mouthpiece of a manometer at 40 mm Hg for 15 s, and the ratio of the longest R-R interval to the shortest R-R interval during the test was calculated; a ratio below 1.10 was considered abnormal^1,12^. Based on the criteria proposed by the Toronto Diabetic Neuropathy Expert Group, early and definite involvements of cardiovascular autonomic dysfunction were identified as one abnormal and at least two abnormal HR test results, respectively^13^. In the current study, CAN was defined as definite involvement of cardiovascular autonomic dysfunction^13^. Individuals were advised to avoid caffeinated beverages, smoking, and alcohol for the 12 h preceding the cardiovascular tests and avoid taking the drugs, including antihistamines, diuretics, and beta-blockers, for two days before the tests. Three patients were excluded as they could not perform one or more of the autonomic function tests. In total, 287 subjects were included in this study.

### Statistical analysis

Data are presented as means ± standard deviations (SDs) or frequencies (percentages), unless otherwise remarked. Categorical variables were analyzed using the chi-squared test, and the Mann–Whitney U test or Student's t-test was used for continuous variables. Data with a skewed distribution were logarithmically transformed before regression analysis. In order to compare mean S1P levels according to the degree of cardiovascular autonomic dysfunction, analysis of covariance was performed after adjusting for confounding factors. Using logistic regression models, multivariable analysis adjusting for identified factors and previously known risk factors was conducted to assess the association between S1P levels and CAN. The use of insulin and oral hypoglycemic agents (OHAs) was coded as a dummy variable. A multivariable model with CAN as the dependent variable was used to investigate interactions between S1P level and other covariates. When a significant interaction was identified between S1P and any of the covariates (*p* for interaction < 0.05), stratified analysis was then performed. A significant *p* value of < 0.013 was used to correct for multiple comparisons. All statistical analyses were conducted using SPSS version 20.0 (SPSS, Chicago, IL, USA). An α level of 0.05 was used for statistical tests.

## Results

Of the total 287 participants, 149 (51.9%) subjects were men. The prevalence of CAN was 25.1% (n = 72). The clinical characteristics of individuals with type 2 DM according to the presence of CAN are presented in Table 1. Individuals with CAN had a longer duration of diabetes, were older, and had a higher prevalence of hypertension and nephropathy than those without CAN. Plasma S1P concentrations were significantly lower in individuals with CAN than in those without CAN.

**Table 1:** Characteristics of individuals with type 2 diabetes.

	CAN (–)	CAN ( +)	*p* value
n	215	72	
Age (years)	58.6 ± 12.6	63.7 ± 9.6	< 0.001
Men (%)	108 (50.2)	41 (56.9)	0.324
Current smoker, n (%)	31 (14.4)	14 (19.4)	0.310
Hypertension, n (%)	110 (51.2)	49 (68.1)	0.013
Hyperlipidemia, n (%)	123 (57.2)	39 (54.2)	0.652
Diabetes duration (years)	3.0 (0.2–10.0)	10.0 (2.0–20.0)	< 0.001
Body mass index (kg/m^2^)	25.9 ± 4.4	25.6 ± 3.3	0.584
Systolic blood pressure (mmHg)	135.4 ± 18.5	136.6 ± 18.9	0.635
Diastolic blood pressure (mmHg)	78.8 ± 12.6	75.8 ± 12.2	0.106
A1C (%)	7.8 ± 1.8	8.0 ± 1.9	0.429
A1C (mmol/mol)	62 ± 20	64 ± 21	0.429
Total cholesterol (mmol/l)	4.4 ± 1.2	4.3 ± 1.1	0.435
Triglyceride (mmol/l)	1.3 (1.0–2.0)	1.4 (1.1–1.8)	0.374
HDL-C (mmol/l)	1.3 ± 0.3	1.2 ± 0.4	0.867
LDL-C (mmol/l)	2.6 ± 0.9	2.5 ± 0.8	0.298
hs-CRP (mg/dl)	0.08 (0.03–0.14)	0.06 (0.03–0.13)	0.475
UAER (mg/gCr)	12.9 (7.3–38.0)	14.8 (8.3–105.8)	0.216
eGFR (ml/min/1.73m^2^)	96.5 (85.7–106.1)	93.5 (71.4–100.8)	0.016
S1P (µmol/l)	9.5 (7.2–12.0)	8.1 (6.4–11.2)	0.024
Retinopathy, n (%)	42 (19.5)	20 (27.8)	0.141
Nephropathy, n (%)	25 (11.6)	17 (23.6)	0.013
Use of lipid-lowering agents, n (%)	106 (49.3)	34 (47.2)	0.760
Use of oral hypoglycemic agents, n (%)	131 (60.9)	50 (69.4)	0.195
Use of insulin, n (%)	25 (11.6)	12 (16.7)	0.269
Use of ACE inhibitors/ARBs, n (%)	80 (37.2)	34 (47.2)	0.133
Use of β-blockers, n (%)	18 (8.4)	10 (13.9)	0.172
Use of diuretics, n (%)	23 (10.7)	12 (16.7)	0.180

Values are presented as the mean ± standard deviation or median (interquartile range). Statistical testing by the chi-squared test, the Mann–Whitney U test or Student's t-test. A1C, glycated hemoglobin; ACE, angiotensin-converting enzyme; ARB, angiotensin II receptor blocker; CAN, cardiovascular autonomic neuropathy; eGFR, estimated glomerular filtration rate; HDL-C, high density lipoprotein cholesterol; hs-CRP, high-sensitivity C-reactive protein; LDL-C, low density lipoprotein cholesterol; S1P, Sphingosine 1-phosphate; UAER, urinary albumin excretion rate.

**Figure 1:**
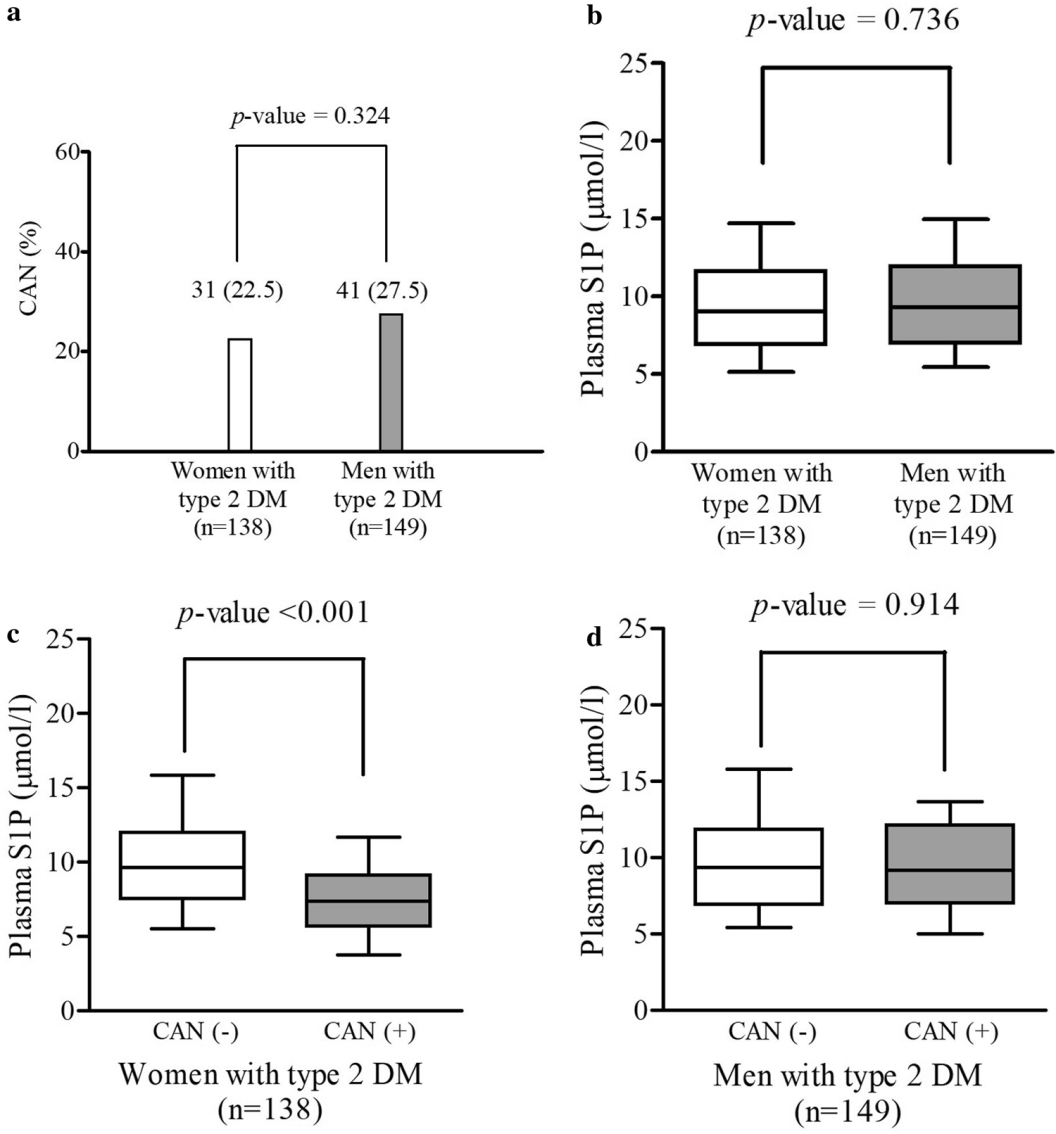
Characteristics of individuals with type 2 diabetes according to sex. (**A**) Prevalence of CAN according to sex. (**B**) Plasma S1P levels according to sex. (**C**) Plasma S1P levels in women according to CAN. (**D**) Plasma S1P levels in men according to CAN. Data are represented as frequencies (percentages). Statistical testing by the chi-squared test or the Mann–Whitney U test. Horizontal bars represent the 10th, 25th, 50th, 75th, and 90th percentile levels. CAN, cardiovascular autonomic neuropathy; S1P, sphingosine 1-phosphate.

A significant interaction was identified between S1P levels and sex (*p* = 0.003) (Supplementary Table1), indicating that the effect of S1P on CAN may be affected by sex. To further evaluate the interaction between S1P and sex, we conducted stratified analysis according to sex. There was no difference in the prevalence of CAN and plasma S1P levels between women and men with type 2 DM (women 22.5% vs. men 27.5%, *p* = 0.324; women 9.0 µmol/l vs. men 9.3 µmol/l, *p* = 0.736, respectively, Fig. 1 and Supplementary Table 2). When stratified by sex, plasma S1P concentrations were significantly lower in women with CAN than in those without CAN (no CAN 9.6 µmol/l vs. CAN [ +] 7.4 µmol/l, *p* < 0.001), but there was no significant difference in plasma S1P levels between men with and without CAN (no CAN 9.4 µmol/l vs. CAN [ +] 9.2 µmol/l, *p* = 0.914) (Fig. 1). Average plasma S1P concentrations according to the degree of cardiovascular autonomic dysfunction in women and men are presented in Table 2. In women, plasma S1P levels significantly differed according to the degree of cardiovascular autonomic dysfunction, after adjusting for age, body mass index (BMI), HDL-C, LDL-C, triglycerides, hypertension, A1C, high-sensitivity C-reactive protein (hs-CRP), diabetes duration, retinopathy and nephropathy (normal, 9.8 μmol/l, 95% CI 8.9–10.9; early, 9.0 μmol/l, 95% CI 8.0–10.0; definite, 7.1 μmol/l, 95% CI 6.2–8.1; *p* for trend = 0.002). In men, however, there was no significant difference in plasma S1P levels according to the degree of cardiovascular autonomic dysfunction.

**Table 2:** Comparison of plasma S1P levels according to the severity of cardiovascular autonomic dysfunction.

Plasma S1P (µmol/l)		Degree of cardiovascular autonomic dysfunction			*p* for trend
		Normal	Early	Definite	
Women	Model 1	9.8 (8.9–10.8)	8.9 (8.0–9.9)	7.2 (6.3–8.3)	0.003
	Model 2	9.9 (8.9–10.9)	8.9 (8.0–9.9)	7.1 (6.1–8.1)	0.001
	Model 3	9.8 (8.9–10.9)	9.0 (8.0–10.0)	7.1 (6.2–8.1)	0.002
Men	Model 1	9.4 (8.4–10.6)	8.7 (7.7–9.7)	9.4 (8.2–10.8)	0.524
	Model 2	9.6 (8.6–10.7)	8.4 (7.5–9.4)	9.6 (8.5–11.0)	0.165
	Model 3	9.6 (8.6–10.9)	8.4 (7.5–9.5)	9.8 (8.5–11.3)	0.167

S1P, sphingosine 1-phosphate.

^a^Values are presented as geometric mean (95% confidence interval).

Model 1: adjusted by age.

Model 2: adjusted by model 1 plus body mass index, HDL-C, LDL-C, triglycerides^a^, and hypertension.

Model 3: adjusted by model 2 plus A1C, hs-CRP^a^, diabetes duration^a^, retinopathy, and nephropathy.

Using logistic regression models, the association between plasma S1P levels and CAN was investigated in women and men with type 2 DM (Table 3). In women, the relationship between plasma S1P levels and CAN was statistically significant (odds ratio [OR] per SD increase in the log-transformed value, 0.40; 95% CI, 0.23–0.70, *p* = 0.001) after adjustment for age, BMI, HDL-C, LDL-C, triglycerides, hypertension, A1C, hs-CRP, diabetes duration, retinopathy, and nephropathy (model 3). In women, further adjustment for menopausal status did not alter the results (OR per SD increase in the log-transformed value, 0.39; 95% CI, 0.22–0.69, *p* = 0.001). However, no significant relationship was identified between plasma S1P levels and CAN in men. Alternatively, when hyperlipidemia and the use of insulin and OHAs were included as independent variables in the models and HDL-C, LDL-C, and triglycerides were excluded, plasma S1P levels were still associated with CAN in women (Supplementary Table 3).

**Table 3:** Logistic regression models of the association between S1P levels and cardiovascular autonomic neuropathy in individuals with type 2 diabetes according to sex.

Plasma S1P^a^ (µmol/l)	Cardiovascular autonomic neuropathy					
	Women			Men		
	OR	95% CI	*p* value	OR	95% CI	*p* value
Unadjusted	0.43	0.27–0.69	< 0.001	1.01	0.71–1.43	0.971
Model 1	0.46	0.28–0.75	0.002	1.10	0.76–1.60	0.617
Model 2	0.43	0.25–0.72	0.001	1.20	0.80–1.82	0.379
Model 3	0.40	0.23–0.70	0.001	1.25	0.78–1.99	0.355

CI, confidence interval; OR, odds ratio; S1P, sphingosine 1-phosphate.

^a^Values were log-transformed prior to analysis.

Model 1: adjusted by age.

Model 2: adjusted by model 1 plus body mass index, HDL-C, LDL-C, triglycerides^a^, and hypertension.

Model 3: adjusted by model 2 plus A1C, hs-CRP^a^, diabetes duration^a^, retinopathy and nephropathy.

## Discussion

In the current study, we investigated the relationship between plasma S1P levels and CAN in individuals with type 2 DM. A significant interaction was identified between plasma S1P levels and sex with respect to CAN. When stratified by sex, the association between plasma S1P levels and CAN was observed only in women; plasma S1P levels were significantly and inversely associated with CAN in women after adjustment for well-known risk factors including A1C, DM duration, and hypertension. In addition, plasma S1P levels might be related to the severity of CAN in women with type 2 DM.

Recently, S1P has emerged as a bioactive signaling molecule, which influences a diverse range of cellular functions by acting as an extracellular signal or an intracellular second messenger^5^. S1P is much more abundant in blood than most tissues in which it is degraded via S1P lyase or S1P phosphatases^14,15^. It has been suggested that the majority of S1P signaling occurs through binding to cell surface receptors^16^. S1P receptors are widely expressed in many tissues, particularly in the nervous, cardiovascular, and immune systems^17^. Therefore, S1P has been suggested as an easily accessible biomarker in clinical settings^8^.

Previous investigations have reported that S1P may protect against the development of diabetes^18–21^. Previous clinical studies also showed that S1P levels were lower in patients with type 2 DM compared with those without type 2 DM^22,23^. In addition, accumulating evidence has demonstrated that S1P has crucial signaling functions in the nervous system^6^. Experimental studies have shown that S1P plays a pivotal role in neural development and may regulate neuronal differentiation, survival, excitability, arborization, and calcium signaling^6,24,25^. S1P was also found to be involved in neuronal repair^6^. Preclinical studies reported that administration of a S1P receptor agonist improved neuronal electrophysiologic function and reduced demyelination^26,27^. Szepanowski et al.^28^ also reported that treatment with a S1P receptor agonist enhanced axonal outgrowth after nerve injury, suggesting a beneficial role of S1P during the nerve regeneration process. In addition, reduced levels of S1P may be implicated in degenerative disorders in the nervous system^8^. Therefore, findings from previous studies have suggested that S1P may play a physiological neuroprotective role. However, the relationship between S1P and cardiovascular autonomic dysfunction remains unclear. To our knowledge, this is the first study to explore the relationship between plasma S1P levels and CAN in individuals with type 2 DM. Our results showed a significant and inverse relationship between plasma S1P levels and CAN in individuals, particularly women, with type 2 DM after adjustment for well-known risk factors for CAN. This suggests that S1P may be protective against cardiovascular autonomic dysfunction in type 2 DM patients, although the causal nature of this association could not be confirmed in this study. Plasma S1P pool is suggested to be bound to HDL-C, LDL-C, and very low-density lipoproteins^29^. Several studies have reported that dyslipidemia is associated with CAN^3,30^. Thus, these lipoprotein abnormalities might contribute, in part, to the relationship between S1P and CAN in the current study. However, in the multivariable analysis, an association between S1P levels and CAN remained statistically significant, after adjustment for confounding factors including HDL-C, LDL-C, and triglycerides, indicating that these factors did not significantly influence the association between S1P and CAN.

Although the mechanisms by which S1P is linked to CAN remain unclear, plausible explanations exist. S1P is related to oxidative stress^31^ and endothelial function^8^, and S1P is known to decrease oxidative stress in neuronal cells^31^. Furthermore, S1P is thought to enhance endothelial barrier function, and to be involved in the maintenance of vascular integrity and the repair of vascular injury^8^. Chronic hyperglycemia results in oxidative stress, and increased oxidative stress induces neuronal damage^1^. Endothelial damage also impairs neuronal blood flow^32^. These pathways are considered to be critical contributors to the pathogenesis of autonomic neuropathy^32,33^. In addition, S1P is involved in inflammation and immune reactions^34^. Therefore, even though the causal relationships could not be established in this study, these findings indicate that S1P plays a protective role in the pathogenesis of CAN.

In the present study, the association between S1P levels and CAN in individuals with type 2 DM showed a sex difference. Although the reason for this sex difference remains unclear, sex hormones may underlie this sex-specific association between S1P levels and CAN. Several studies have suggested that estrogen might modulate the autonomic nervous system^35^. Estrogen treatment has previously been reported to decrease sympathetic drive and ameliorate cardiac autonomic control^36^. Furthermore, in vitro studies, estrogen has been shown to enhance the synthesis and export of S1P from cancer cell lines^37^. In the study of 108 healthy participants, Guo et al.^38^ reported that plasma S1P levels were higher in women than in men. In our study, however, there was no significant difference in the prevalence of CAN between women and men, which correlates with previous studies^39,40^. Unlike the findings reported by Guo et al.^38^, in this study, plasma S1P levels did not significantly differ between women and men with type 2 DM, which may be a result of differences in study populations. Moreover, in our study, menopausal status did not significantly affect the relationship between plasma S1P levels and CAN in women. Thus, it suggests that additional mechanisms might exist. HDL may be in part implicated in a sex-specific association between S1P levels and CAN. Previous studies have suggested that higher levels of HDL particles are observed in women compared with men^41,42^. HDL function is partly dependent on S1P. HDL-associated S1P may contribute to the anti-oxidative and anti-inflammatory functions of HDL, which relate to protective roles against vascular damage^43,44^. Additionally, a sex difference might be assumed to be due to adverse impacts of androgen in men, although it is currently unproven^45^. Further studies are therefore required to explore the mechanism underlying the sex difference in association between S1P levels and CAN.

We note that ceramide promotes apoptosis, which is linked to an opposing action to S1P^5^. In an experimental study, apoptosis of mouse Schwann cells was enhanced by incubation with palmitate, and these effects were suppressed by inhibition of serine-palmitoyl transferase, the rate-limiting step of ceramide biosynthesis, suggesting a potential role of ceramide in nerve damage^46^. In contrast, Dohrn et al.^47^ found no significant differences in plasma levels of the ceramide precursor sphinganine, including C16-, C17-, C18-, and C20-sphinganine, between type 2 DM patients with distal sensorimotor polyneuropathy and healthy controls. Therefore, future studies to investigate the role of ceramide in CAN would provide additional information to better understand pathogenic mechanisms of S1P in cardiovascular autonomic dysfunction.

There are some limitations in our study. First, because of the cross-sectional nature of the study, the causal relationships could not be ascertained. Second, although mass spectrometry-based assay is suggested as the gold standard technique for sphingolipid analysis^48^, the method using commercial ELISA kits has been increasingly used to measure S1P level^10,49,50^. In addition, this method used in our study was previously validated by liquid chromatography-tandem mass spectrometry^51^. Finally, although each of HR variability tests has good sensitivity and specificity of ~ 90%^1^ and abnormal HR variability in one test indicates early autonomic neuropathy^1,4^, these tests might not be able to detect abnormalities in early subclinical CAN. Thus, early involvement of cardiovascular autonomic dysfunction might be underestimated in this study. However, because this would bias toward the null, we believe that our findings would not be significantly affected. Despite these limitations, our data might give an important information with regard to relationships between plasma S1P levels and CAN in type 2 DM patients.

In conclusion, we identified an inverse relationship between plasma S1P levels and CAN only in women with type 2 DM. Further studies are necessary to determine the mechanism underlying the sex difference in the association between S1P levels and CAN.

## Supplementary information

Supplementary Information.
