# Supplementary material for: Plasma sphingosine 1-phosphate concentrations and cardiovascular autonomic neuropathy in individuals with type 2 diabetes
Source: Sci Rep. 2020 Jul 29;10:12768. doi: 10.1038/s41598-020-69566-y (PMC7391653; doi:10.1038/s41598-020-69566-y)
Supplement: Supplementary file 1 — Supplementary Information. [file 41598_2020_69566_MOESM1_ESM.pdf]

Plasma sphingosine 1-phosphate concentrations and cardiovascular autonomic neuropathy in individuals with type 2 diabetes

Min Young Chung[1], Seon-Young Park[2], Jin Ook Chung[1], Dong Hyeok Cho[1], Dong Jin Chung[1]

[1]Division of Endocrinology and Metabolism, Department of Internal Medicine, Chonnam National University Medical School, Gwangju, Republic of Korea; [2]Division of Gastroenterology and Hepatology, Department of Internal Medicine, Chonnam National University Medical School, Gwangju, Republic of Korea

Supplementary Table 1. Logistic regression models predicting cardiovascular autonomic neuropathy

	Unadjusted		Adjusted[a]	
	OR (95% CI)	*p*-value		*p*-value
Sex (male)	1.31 (0.77–2.24)	0.324	0.10 (0.02–0.55)	0.008
S1P	0.72 (0.55–0.95)	0.019	0.57 (0.40–0.81)	0.001
S1P*sex				0.003

[a]This model adjusted for age, body mass index, LDL-C, HDL-C, triglycerides, hypertension, A1C, hs-CRP, diabetes duration, retinopathy, and nephropathy

CI, confidence interval; OR, odds ratio; S1P, sphingosine 1-phosphate

Supplementary Table 2. Characteristics of individuals with type 2 diabetes according to sex

	Women	Men	*p*-value
n	138	149	
CAN, n (%)	31 (22.5)	41 (27.5)	0.324
Age (years)	62.0 ± 12.1	57.8 ± 11.8	0.003
Hypertension, n (%)	80 (58.0)	79 (53.0)	0.399
Hyperlipidemia, n (%)	74 (53.6)	88 (59.1)	0.353
Diabetes duration (years)	4.0 (0.5–13.3)	3.3 (0.3–12.0)	0.618
Body mass index (kg/m^2)	25.7 ± 4.8	26.0 ± 3.5	0.604
Systolic blood pressure (mmHg)	136.3 ± 18.8	135.1 ± 18.4	0.575
Diastolic blood pressure (mmHg)	76.3 ± 11.8	79.4 ± 13.0	0.033
A1C (%)	7.6 ± 1.8	8.0 ±1.9	0.066
A1C (mmol/mol)	60 ± 19	64 ± 21	0.066
Total cholesterol (mmol/l)	4.5 ± 1.2	4.3 ± 1.1	0.142
Triglyceride (mmol/l)	1.3 (0.9–1.8)	1.4 (1.0–2.1)	0.028
HDL-C (mmol/l)	1.3 ± 0.3	1.2 ± 0.3	<0.001
LDL-C (mmol/l)	2.7 ± 0.9	2.5 ± 0.9	0.260
hs-CRP (mg/dl)	0.06 (0.03–0.14)	0.08 (0.03–0.13)	0.321
UAER (mg/gCr)	13.0 (8.2–33.1)	14.4 (6.6–48.1)	0.738
eGFR (ml/min/1.73m^2)	95.8 (85.0–104.5)	95.2 (79.2–104.7)	0.630
S1P (μmol/l)	9.0 (6.9–11.6)	9.3 (7.0–11.9)	0.736
Retinopathy, n (%)	30 (21.7)	32 (21.5)	0.957
Nephropathy, n (%)	17 (12.3)	25 (16.8)	0.286
Menopause, n (%)	111 (80.4)	–	–
Use of lipid-lowering agents, n (%)	65 (47.1)	75 (50.3)	0.584
Use of oral hypoglycemic agents, n (%)	83 (60.1)	98 (65.8)	0.324
Use of insulin, n (%)	21 (15.2)	16 (10.7)	0.258
Use of ACE inhibitors/ARBs, n (%)	59 (42.8)	55 (36.9)	0.312
Use of β-blocker, n (%)	11 (8.0)	17 (11.4)	0.327
Use of diuretics, n (%)	18 (13.0)	17 (11.4)	0.673

Values are presented as the mean ± standard deviation or median (interquartile range). A1C, glycated hemoglobin; ACE, angiotensin-converting enzyme; ARB, angiotensin II receptor blocker; CAN, cardiovascular autonomic neuropathy; eGFR, estimated glomerular filtration rate; HDL-C, high density lipoprotein cholesterol; hs-CRP, high-sensitivity C-reactive protein; LDL-C, low density lipoprotein cholesterol; S1P, Sphingosine 1-phosphate; UAER, urinary albumin excretion rate

Supplementary Table 3. Logistic regression models of the association between S1P levels and cardiovascular autonomic neuropathy when hyperlipidemia and anti-diabetic therapy were included as independent variables and HDL-C, LDL-C, and triglycerides were excluded

Plasma S1P† (μmol/l)	Cardiovascular autonomic neuropathy: Women			Men		
	OR	95% CI	*p*-value	OR	95% CI	*p*-value
Unadjusted	0.43	0.27–0.69	<0.001	1.01	0.71–1.43	0.971
Model 1	0.46	0.28–0.75	0.002	1.10	0.76–1.60	0.617
Model 2	0.45	0.27–0.74	0.002	1.14	0.77–1.68	0.515
Model 3	0.43	0.25–0.74	0.002	1.24	0.78–1.96	0.358

CI, confidence interval; OR, odds ratio; S1P, sphingosine 1-phosphate

†Values were log-transformed prior to analysis.

Model 1: adjusted by age

Model 2: adjusted by model 1 plus body mass index, hyperlipidemia, and hypertension

Model 3: adjusted by model 2 plus A1C, hs-CRP†, diabetes duration†, retinopathy, nephropathy, and use of OHAs and insulin
